# Biofilm Formation on the Surface of (Poly)Ether-Ether-Ketone and In Vitro Antimicrobial Efficacy of Photodynamic Therapy on Peri-Implant Mucositis

**DOI:** 10.3390/polym13060940

**Published:** 2021-03-18

**Authors:** Tzu-Yu Peng, Dan-Jae Lin, Yuichi Mine, Chi-Yang Tasi, Po-Jung Li, Yin-Hwa Shih, Kuo-Chou Chiu, Tong-Hong Wang, Shih-Min Hsia, Tzong-Ming Shieh

**Affiliations:** 1 School of Dentistry, College of Dentistry, China Medical University, Taichung 40402, Taiwan; typ@mail.cmu.edu.tw (T.-Y.P.); djlin@mail.cmu.edu.tw (D.-J.L.); ll820731@gmail.com (P.-J.L.); 2 Department of Medical System Engineering, Graduate School of Biomedical and Health Sciences, Hiroshima University, Hiroshima City, Hiroshima 734-8553, Japan; mine@hiroshima-u.ac.jp; 3 Department of Dentistry, Taipei Medical University Hospital, Taipei 11031, Taiwan; cytsai@tmu.edu.tw; 4 Department of Healthcare Administration, College of Medical and Health Science, Asia University, Taichung 41354, Taiwan; evashih@asia.edu.tw; 5 Division of Oral Diagnosis and Family Dentistry, Tri-Service General Hospital, National Defense Medical Center, Taipei 11490, Taiwan; 6 Tissue Bank, Chang Gung University, Chang Gung Memorial Hospital, Taoyuan 33305, Taiwan; cellww@adm.cgmh.org.tw; 7 School of Nutrition and Health Sciences, Taipei Medical University, Taipei 11031, Taiwan; bryanhsia@tmu.edu.tw

**Keywords:** dentistry, polyetheretherketone, biofilm, peri-implant mucositis, photodynamic therapy, antimicrobial activity

## Abstract

Poly-ether-ether-ketone (PEEK) is an aesthetically pleasing natural material with good biocompatibility and shock absorption characteristics. The application of PEEK as a dental implant or abutment is expected to reduce the risk of failure and enhance aesthetics. Given that approximately one in 15 patients have allergic reactions to antibiotics, photodynamic therapy (PDT) has been gaining attention as an alternative treatment. Herein, the applicability of PEEK dental implants or abutments was investigated using material analyses, biofilm formation assay, and cell viability tests. The possible use of PDT for peri-implant mucositis was evaluated with the biofilm removal assay. The obtained data were analyzed based on the multivariate analysis of variance, paired t-tests, and the Pearson correlation coefficient (α = 0.05). The results revealed that PEEK was significantly less conducive to the formation of biofilms with *S. mutans* and *A. actinomycetemcomitan (p* < 0.001) but exhibited comparable MG-63 (human osteoblast-like) osteoblast cell viability (*p* > 0.05) to the other materials. PDT had similar antimicrobial efficacy and yielded similar biofilm removal effects to antibiotics. Altogether, these findings suggest that PEEK has attractive features and can serve as an alternative material for dental implants or abutments. In cases where peri-implant mucositis occurs, PDT can be used as an accessible therapeutic approach.

## 1. Introduction

Given the increasing importance of aesthetic concepts in contemporary society, aesthetic rehabilitation is gradually becoming more prominent [1]. Correspondingly, the oral medical system has undergone unprecedented changes. Among them, dental implantology is one of the most eye-catching therapeutic approach choices, which not only successfully meets aesthetic considerations, but expands the applicability of various types of dental prostheses (i.e., all-on-four implant therapy, overdentures, etc.) [2,3,4].

Titanium alloys are currently considered as the gold standard materials for dental implants or abutments owing to their good biocompatibility [5,6]. However, the metallic color of the titanium implant or abutment is visible through the soft peri-implant tissue, and this results in an unpleasant aesthetic outcome [7]. The long-term existence of metal materials in humid oral environments can easily lead to chemical corrosion [8]; additionally, patients with metallic allergies will have a greater risk of developing allergic reactions to metals or alloy materials [9]. Therefore, in the past decade, the choice of materials for dental implants or abutment has gradually shifted to aesthetic materials. Evidence from several studies pointed out that compared with titanium alloys, the aesthetic zirconia-based ceramic materials demonstrated a low risk of inflammatory reactions and enhanced bone apposition; correspondingly, zirconia is viewed as a new dental implant or abutment material [10,11,12]. Nonetheless, the hardness characteristics will allow zirconia implants or abutments to directly transfer the stresses or impact forces (e.g., occlusal forces) to the surrounding bones via the implant–bone interface, and thus impose a heavy burden on the jawbone and induce other undesirable symptoms [13,14]. Thus, the clinical applicability of zirconia to dental implants or abutments still generates concern.

Poly-ether-ether-ketone (PEEK) materials with natural and aesthetic properties recently attracted considerable attention in the field of dentistry [15]. PEEK has similar mechanical properties to human bone; thus, when PEEK is used for implants or abutments, it can serve as a good shock absorption material that can spread the occlusion force and transfer the stress to the bone smoothly so as to avoid the occurrence of failure [16,17]. The literature had reported that PEEK was beneficial to prolong the implant lifespan [18]. Regarding the application of PEEK in dental practice, some scholars had proposed PEEK frameworks veneered with composite resin as a solution for dental prostheses for patients with metal allergies [19,20]. Other reports indicated using PEEK as the framework combined with gingival composite resin and lithium disilicate crowns to rehabilitate the mandible dentition for edentulous patients [21]. The feasibility of the above clinical applications was based on the excellent material properties of the PEEK, e.g., lighter weight, higher elasticity, and aesthetics. Therefore, PEEK can reduce the risk of metal allergies and mechanical complications. Although dental implants (screw) fabricated by PEEK have not been extensively used nowadays clinically [22], PEEK is supposed to be an alternative to conventionally applied implant or abutment materials, such as titanium and zirconia.

The definition of success for the implant (or abutment) is not limited to implant survival, but includes the maintenance of the health of peri-implant tissues despite the complex microbial challenge in the oral environment [23]. Similar to titanium or zirconia, if bacterial challenges are presented and excessive host responses are evoked when PEEKs are used, peri-implant mucositis will occur. If peri-implant mucositis cannot be reduced, it will lead to peri-implantitis and a series of unexpected symptoms. In light of this, the inception and implementation of successful efforts to reduce peri-implant mucositis are critical. The standard nonsurgical therapy for peri-implant mucositis in the dental clinic is the administration of antibiotics [23,24]. However, according to a statistical report from the National Health Service (NHS, UK), approximately one in 15 patients have an allergic reaction to antibiotics [25]. Thus, the focus has recently shifted toward mechanical therapy, such as phototherapy with laser, infrared, or other alternative light sources [7]. Photodynamic therapy (PDT) is an elegant light-based intervention technique [26]. The PDT mechanism involves the use of a photosensitizer that is activated by exposure to light of a specific wavelength (usually illuminated with a long wavelength of visible red light, i.e., 620–690 nm) in the presence of oxygen [27]. The light activates the photosensitizer to transform from a low-energy state to a high-energy state [7,27]. In aerobic conditions, this leads to the generation of cytotoxic species, and consequently results in cell death and tissue devastation [28]. The photosensitizer concentrates within the infected tissue. Thus, when the light is directly focused on the lesion, it will generate reactive oxygen species that will lead to cellular destruction [28]. Because PDT can be applied to a wide variety of different diseases, it has recently become an alternative therapy in the medical field [27,29,30]. Evidence from other studies indicated that PDT represents a novel therapeutic approach in managing oral biofilms and that it can be used as an adjunct antimicrobial protocol for peri-implant mucositis [7,27]. For this reason, this study considers PDT as a therapeutic approach for peri-implant mucositis.

To the best of our knowledge, only a few studies have discussed the biofilm formation and removal assays on the surface of PEEK. More trials are vital to evaluate whether PEEK materials have lower biofilm formation ability suitable for implants or abutments, and whether PDT can replace antibiotics as a therapeutic approach for peri-implant mucositis. Herein, the present in vitro studies have two goals: (1) investigating the biofilm formation abilities and MG-63 osteoblast cell viability responses of PEEK in compared with other biomaterials, and (2) evaluating the antimicrobial efficacy of PDT.

## 2. Materials and Methods

### 2.1. Sample Preparation and Surface Treatment

**Figure 1:**
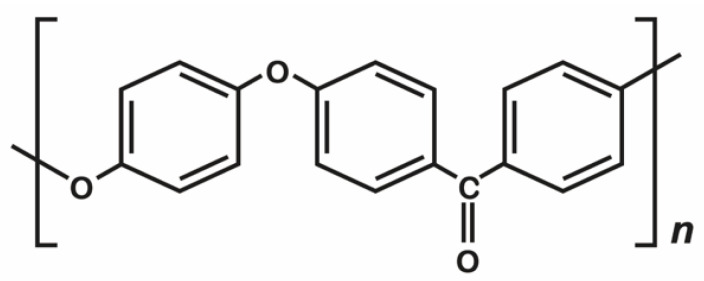
The chemical structure of PEEK.

Three different types of dental materials were considered: alloy material—titanium grade 5 (Ti-6Al-4V), ceramic material—yttria-stabilized tetragonal zirconia polycrystal (Y-TZP), and polymer material—PEEK (Figure 1). The details on the materials used in this study are listed in Table 1. All testing samples were disk-shaped with a diameter of 10 mm and thickness of 2.5 mm. Grinding was used as the surface treatment method. All the testing samples were ground with silicon carbide abrasive paper in the order of #600, #1000, and #1500 to make the surface smooth, followed by ultrasonic cleaning (RUC-101, REXMED Industries Co., Ltd., Kaohsiung, Taiwan) with air drying. All the samples were immersed in deionized water at 37 °C for 24 h prior to the experiment.

### 2.2. Surface Roughness and Morphologies

The surface roughness (Ra) of the samples prepared for each type of material (*n* = 5) was measured as the arithmetic mean deviation of the profile obtained with the use of a stylus profilometer (DektakXT, Bruker Taiwan Co., Ltd., Hsinchu, Taiwan). Three different regions were evaluated in each sample to determine 3 Ra values, and the average values were estimated to characterize the final Ra value of each sample. The representative surfaces were observed by a thermal field emission scanning electron microscope (Thermal FE-SEM; JEOL JSM-7800F Prime, JEOL Ltd., Tokyo, Japan). The specimen chamber was set at a vacuum level of 9.6 × 10^−5^ Pa, and the specimens were observed under an accelerating voltage of 3.0 kV using secondary electron mode. Images were captured using the lower electron detector of the FE-SEM system, while magnification strengths of 500× and 2000× were used.

### 2.3. Hydrophilicity

The hydrophilicity of the testing samples (*n* = 5) was determined using a contact angle analyzer (FTA-125, First Ten Angstroms, Portsmouth, VA, USA). A droplet of distilled water (~10 μL) was extruded vertically from a 31G needle onto the testing samples at room temperature, and a charge-coupled device (CCD) was triggered to allow continuous image recordings. A non-spherical fitting approach measured the contact angle of each water drop on the picture. Each reported contact angle was the mean of six independent measurements.

### 2.4. Microbial Cultures

All the microbes used in the present study were purchased from Bioresource Collection and Research Center (BCRC) (Hsinchu, Taiwan). *Streptococcus mutans* (*S. mutans*, BCRC number: 10793) and *Aggregatibacter actinomycetemcomitans* (*A. actinomycetemcomitans,* BCRC number: 80375) were used in the study. *S. mutans* was cultured in tryptic soy broth, and *A. actinomycetemcomitans* was cultured in brain heart infusion broth. The bacteria were inoculated by loop transfer from frozen tubes into 3 mL nutrient broth slant and were maintained at 37 °C for 24 h with constant shaking at 200 revolutions per minute (rpm). Bacteria from these cultures were transferred in an appropriate solid medium and incubated overnight. Selected colonies were transferred to an appropriate liquid medium and incubated for 4–6 h to achieve log phase growth. The optical density of each culture at 600 nm (OD 600) was adjusted to 1.0 using fresh broth to obtain a standard inoculum with 1–3 × 10^8^ colony forming units (CFU)/mL [31].

### 2.5. Biofilm Formation Assay

The *S. mutans and A. actinomycetemcomitans* (10^6^ CFU/mL) were inoculated in all the wells of a 24-well plate. The testing samples (Ti-6Al-4V, Y-TZP, and PEEK) were added, mixed well, and cultured at 37 °C for 24, 48, and 72 h. The medium was removed, and the wells were then washed with phosphate-buffered saline (PBS, Life Technologies Limited, Paisley, UK) twice and air dried for 1 h. Crystal violet (150 mL of 0.1% *w*/*v*) was added to each well, and the plate was allowed to remain at room temperature for 10–15 min. The crystal violet was aspirated, and the plate was rinsed three to four times with water. After water aspiration, 150 mL of 33% acetic acid was added to each well. Absorbance was determined at 490 nm (OD 490) using 30% acetic acid in water as the blank on the VersaMax™ ELISA microplate reader (Molecular Device, San Jose, CA, USA) [31].

### 2.6. Biofilm Removal Assay

*S. mutans* and *A. actinomycetemcomitans* (10^6^ CFU/mL) were inoculated per well in a 24-well plate. The testing samples (Ti-6Al-4V, Y-TZP, and PEEK) were added, mixed well, and cultured at 37 °C for 48 h. After the removal of the planktonic bacteria, the biofilms were washed with sterile PBS once. Fifteen samples for each dental material were randomly divided into five groups. To evaluate the removal of pre-existing biofilms, bacteria, 1 mL of PBS containing various doses of temoporfin (0, 1.0, and 2.0 μg/mL), and 100 μg/mL ampicillin were added to each well and incubated for 3 h. The temoporfin and ampicillin treatments were used in conjunction with diode laser (TI-818-1, Transverse Industries Co., Ltd., New Taipei City, Taiwan), a red-light source with emission at 635 nm. The distance from the light to the sample ranged from 3 to 5 cm, and the exposure time was 15 min (10 J/cm^2^). After 4 h of incubation at 37 °C, fixation, staining, and biofilm measurements were performed as described above.

### 2.7. Cell Viability Assay

The cell viability of all tested samples (Ti-6Al-4V, Y-TZP, and PEEK) was measured using the 3-(4,5-dimethylthiazol-2-yl)-2,5-diphenyltetrazolium bromide (MTT) assay. The human osteoblast-like (MG-63) cells (10^5^ cells/1 mL) were inoculated and cultured for 24 h at 37 °C in a humidified atmosphere of 5% CO_2_ in 24-well cell culture plates. To quantify cell viability, 0.5 mL of MTT reagent (M6494, Thermo Fisher Scientific Inc., Waltham, MA, USA) was added to each well and incubated for 4 h at 37 °C. The supernatant was removed, washed with PBS, the blue formazan product was solubilized with 1 mL of dimethylsulfoxide (DMSO, Mediatech. Inc., Manassas, VA, USA), and was finally centrifuged at 150 rpm for 15 min (CN-15, Hsiang Tai Machinery Industry Co., Ltd., New Taipei City, Taiwan). After solubilization, 100 μL of liquid from each well was transferred to a 24-well plate prior to determining absorbance at 590 nm using a VersaMax™ ELISA microplate reader; the obtained data were quantified to determine the cell viability.

### 2.8. Statistical Analysis

The normality of the distribution and the homogeneity of variance were analyzed using the Shapiro-Wilk and Levene’s tests. As all the data exhibited normal distributions, parametric tests were conducted. The comparisons of the results of Ra values and the hydrophilicity were conducted with one-way analysis of variance (ANOVA), and the multiple dental material group (Ti-6Al-4V, Y-TZP, and PEEK) comparisons were analyzed based on Scheffe’s post hoc tests. The correlation between the surface roughness, hydrophilicity, and biofilm formation ability was obtained via Pearson correlation and linear regression. The data obtained from independent biology experiments (biofilm formation ability, antimicrobial assay, and cell viability) with three replicates in each group were analyzed with the paired t-test and one-way ANOVA with post hoc Tukey’s honest significant difference (HSD) tests to compare replicate mean values between different dental material groups in each condition. All statistical analyses were performed with SPSS (version 19, IBM SPSS Statistics, IBM Corporation, New York, NY, USA) (α = 0.05).

## 3. Results

### 3.1. Morphologies, Topography, and Surface Roughness

**Figure 2:**
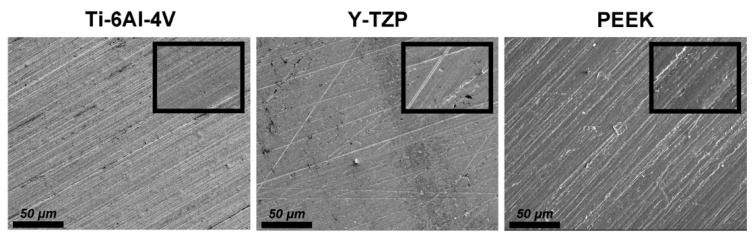
The FE-SEM surface morphologies of each dental materials: the scale bar is 50 μm (magnifications of 500), and the width of the small, enlarged picture is equal to 2.5 μm (magnifications of 2000).

Figure 2 shows the FE-SEM surface morphologies of all tested materials. The surface of the Ti-6Al-4V contained scratch features caused by silicon carbide abrasive paper. These surface characteristics have consistent directionality and are uniform. The surface of Y-TZP also contained some scratch features; however, when compared with other testing materials, the Y-TZP revealed relatively smooth morphologies. PEEK materials had similar surface morphologies with those of the Ti-6Al-4V. However, those features were not as dense as the Ti-6Al-4V, and some polymer impurities had remained on the PEEK surface owing to the surface treatment procedures.

**Figure 3:**
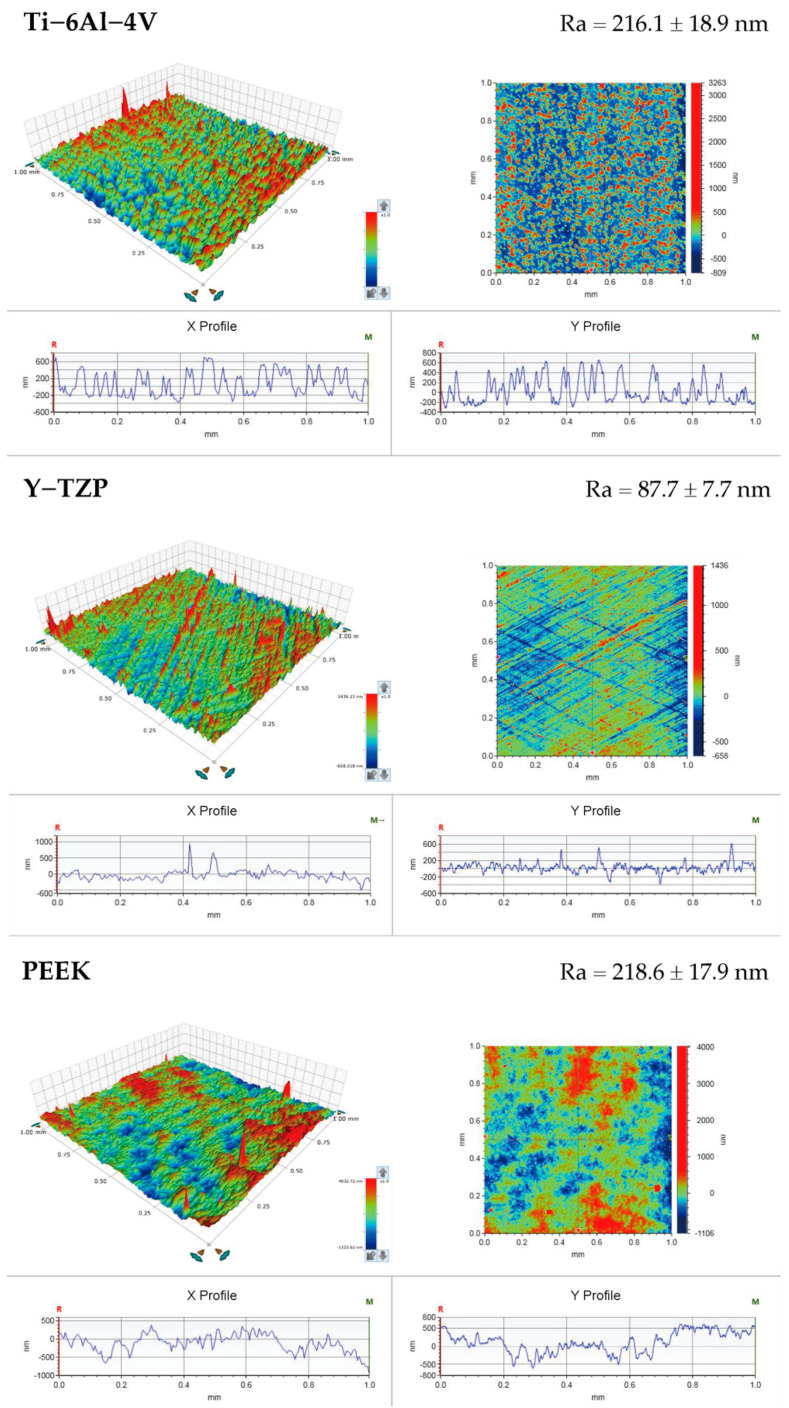
Surface roughness results of each dental materials.

The surface topographies of various materials (Ti-6Al-4V, Y-TZP, and PEEK) are summarized in Figure 3, and the microscale surface roughness (Ra) is shown at the right upper part of each image. The PEEK (218.59 ± 17.95 nm) and the Ti-6Al-4V (216.11 ± 18.85 nm) had comparable Ra values (nonsignificant differences, *p* = 0.696). However, the Y-TZP yielded a significantly lower Ra value (87.66 ± 7.74 nm) compared with the other testing materials (*p* < 0.01).

### 3.2. Hydrophilicity (Contact Angle)

**Figure 4:**
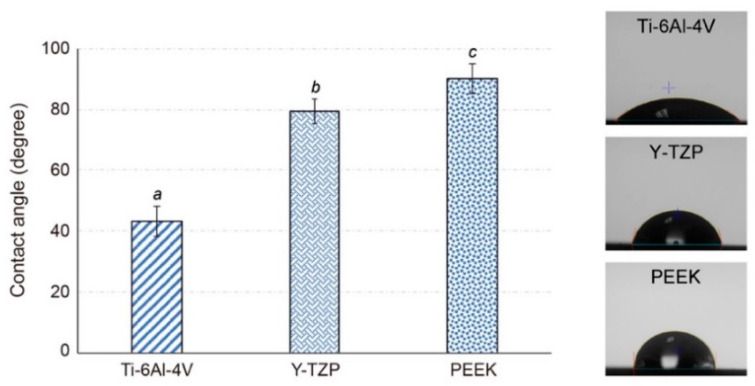
The contact angles results and the representative images of different dental material groups (different letters (a,b,c) indicate groups statistically different, *p* < 0.05).

The levels of hydrophilicity of different tested materials are compared in Figure 4. The contact angles of all the PEEK materials were higher than 90° (90.09° ± 17.95°). This showed a significant super-hydrophobicity feature (*p* < 0.05) compared with the Ti-6Al-4V materials (41.75° ± 18.85°). Although the Y-TZP materials also with high hydrophobicity (79.43° ± 7.74°), the values were still significantly lower than those of PEEK materials (*p* < 0.05).

### 3.3. Biofilm Formation Ability

**Figure 5:**
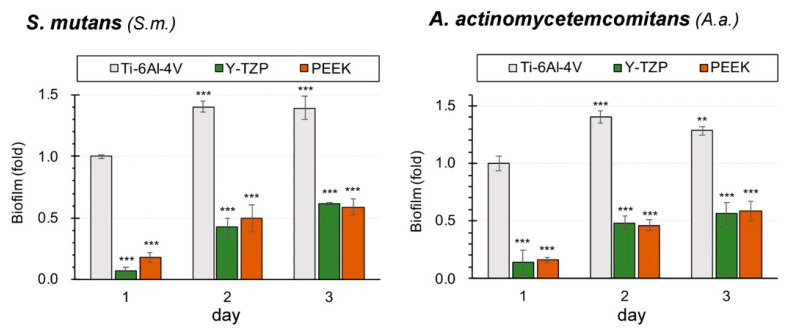
Biofilm formation ability of microbes (significant different to Ti-6Al-4V (day 1), ** *p* < 0.01, *** *p* < 0.001).

Figure 5 illustrates the biofilm formation ability of *S. mutans* and *A. actinomycetemcomitans* on Ti-6Al-4V, Y-TZP, and PEEK. The biofilm formation of the Ti-6Al-4V, regardless of the culture times (24 h, 48 h, or 72 h) or types of bacteria (*S. mutans* or *A. actinomycetemcomitans*), was significantly higher than those of the other testing materials (*p* < 0.001). Among all the materials, the biofilm is associated with a significant growth period from 24 h to 48 h (*p* < 0.001), but the growth was almost stationary after 48 h. It is imperative to note that compared with the Ti-6Al-4V, neither the Y-TZP nor the PEEK were conducive to biofilm formation. According to the Pearson’s correlation analysis, there was a strong negative correlation between the hydrophilicity and biofilm formation ability (Pearson r value was −0.966 for *S. mutans* and −0.977 for *A. actinomycetemcomitans*). The variables of surface roughness and biofilm formation ability yielded a low correlation (Pearson r value < 0.5). Thereby, a larger hydrophilicity (i.e., higher contact angle) was associated with lower biofilm formation ability for identical bacteria.

### 3.4. Biofilm Removal Efficacy

**Figure 6:**
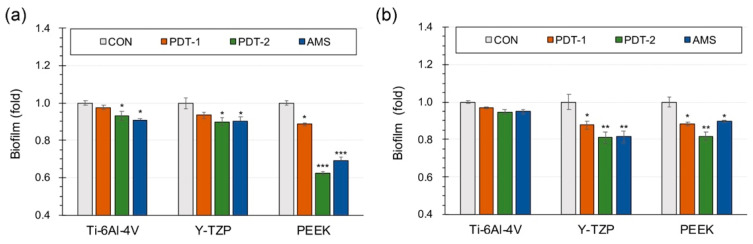
The biofilm removal efficacy of different therapeutic approaches in pre-existing oral microbe biofilms: (**a**) *S. mutans* and (**b**) *A. actinomycetemcomitans*. CON, PDT-1, and PDT-2 correspond to the group using different temoporfin concentrations of 0, 1.0, and 2.0 μg/mL, respectively; AMS is the group using 100 μg/mL ampicillin (significant different to CON group, * *p* < 0.05, ** *p* < 0.01, *** *p* < 0.001).

The biofilm removal assay (Figure 6) revealed that PDT has comparable or better efficacy than that of AMS (the group using 100 μg/mL ampicillin) pertaining to the disruption preformed on *S. mutans* and *A. actinomycetemcomitans* biofilms. In Ti-6Al-4V, PDT with a concentration of 2.0 μg/mL temoporfin (PDT-2) and the AMS group had obvious high-biofilm removal efficacies on *S. mutans* (*p* < 0.05). Nevertheless, PDT with a concentration of 1.0 μg/mL temoporfin (PDT-1) did not demonstrate substantial removal efficacy on preformed biofilms. Moreover, regardless of the therapeutic approaches, there was no effect on the removal of the *A. actinomycetemcomitan* biofilms (*p* > 0.05). All the Y-TZP, PDT-2, and AMS groups exhibited significantly high biofilm removal efficacy on both *S. mutans* and *A. actinomycetemcomitans* (*p* < 0.05) biofilms; however, there was no difference between the PDT-2 and AMS groups. Although PDT-1 exhibited a significant efficacy to disrupt preformed *A. actinomycetemcomitans* biofilms (*p* < 0.05), there was no apparent effect on *S. mutans* biofilms compared with the control group (CON). Regarding the PEEK materials, the PDT-1, PDT-2, and AMS groups all showed significant well biofilm removal efficacy on either *S. mutans* or *A. actinomycetemcomitans* biofilms (*p* < 0.05). Higher temoporfin concentrations were related to better biofilm removal efficacy. Note that the PDT-2 group exhibited the best efficacy pertaining to the disruption of the preformed biofilms (*p* < 0.01).

### 3.5. Cell Viability

**Figure 7:**
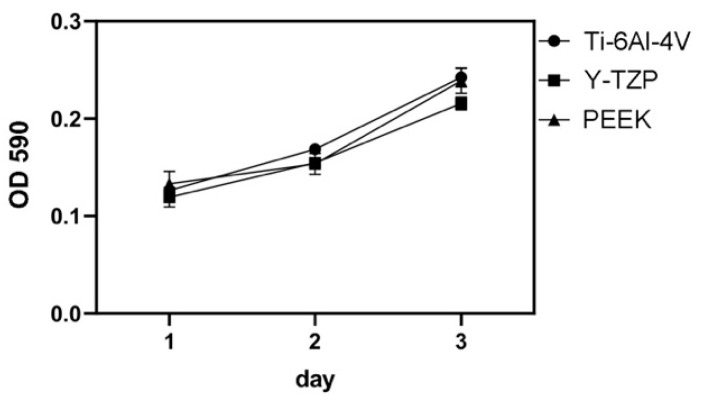
The results of cell viability (MTT test).

The cell viability of MG-63 on each testing dental material (Ti-6Al-4V, Y-TZP, and PEEK) was measured over 1-, 2-, and 3-day periods with the MTT test. The results are shown in Figure 7. Additional comparison indicated that Ti-6Al-4V, Y-TZP, and PEEK yielded similar cell viability outcomes at any time point (*p* > 0.05).

## 4. Discussion

Poly-ether-ether-ketone, commonly referred to as PEEK, is a member of the poly-aryl-ether-ketone polymers (PAEKs) family, and it is a semicrystalline, linear, high-performance thermoplastic polymer which consists of an aromatic molecular chain backbone interconnected by ketone and ether functional groups (Figure 1) [32,33,34]. PEEK confers outstanding mechanical properties, chemical resistance, and an elastic modulus that closely matches natural bone [16]. Furthermore, PEEK can absorb the occlusion force and relieve the occlusion stress and is thus considered as one of the most promising materials for implants or abutments. In this study, we used PEEKs as the target testing materials and titanium alloys (Ti-6Al-4V) and zirconia-based ceramic (Y-TZP) as controls.

The quantitative results of the surface roughness (Ra values) obtained from the stylus profilometer (Figure 3) were consistent with the surface morphology characteristics observed via FE-SEM (Figure 2). The Y-TZP showed a relatively smooth morphology and significantly lower (*p* < 0.001) Ra values after surface treatment compared with other testing materials. This result was in accordance with the literature given that the Vickers hardness of Y-TZP (>1100 Hv) was much higher than those of Ti-6Al-4V (≈300 Hv) and PEEK (≈27 Hv). Thus, the effect of surface treatment was superficial to Y-TZP [13,35]. In addition, the contact angles of PEEKs were approximately 90° (Figure 4), which were significantly higher than those of Ti-6Al-4V and Y-TZP (*p* < 0.001). This was attributed to the fact that the nonpolar functional groups contained in PEEK structures were responsible for the hydrophobicity.

The biofilm formation ability may be influenced by the following two factors: (a) hydrophobicity and hydrophilicity, and (b) surface roughness and surface free energy [36,37]. De-la-Pinta et al. suggested that hydrophilicity was more critical than surface roughness in biofilm formation [38]. The present trial verified similar results that showed that hydrophilicity (Pearson r value > 0.9) was more correlated to the biofilm formation ability than surface roughness (Pearson r value < 0.5). Subject to the same culture conditions, the hydrophobic materials were those which had lower biofilm formation ability, regardless of the types of bacteria (Figure 5). These findings support the fact that the biofilm formation ability of PEEK was lower than that of Ti-6Al-4V. It is known that bacteria can attach and become sheltered in the rougher surfaces of materials [37], thus demonstrating that anti-adhesive action against bacteria would occur on the Y-TZP surface. Although PEEK was significantly more hydrophobic than Y-TZP (*p* < 0.001), the surface roughness of Y-TZP was significantly lower than PEEK (*p* < 0.001). These complex factors indicated that the two materials have comparable biofilm formation abilities.

Gram-positive *S. mutans* are primary and secondary colonizers in dental plaques [39]. The microbiota species around the peri-implantitis sites are very similar to those found in the periodontal disease site, and mainly include the Gram-negative *A. actinomycetemcomitans* [40]. The antibiotic resistance of bacterial cells in biofilms was reported to be 1000 to 1500 times greater than the resistance of planktonic cells. The outer membrane of Gram-negative bacteria plays an important role in relation to its resistance to many antibiotics. Therefore, the elimination of Gram-positive bacteria with PDT is definitely much easier to accomplish than that of Gram-negative bacteria [41]. Therefore, *S. mutans* were more sensitive to *A. actinomycetemcomitan* in both PDT and AMS treatments. In this study, the biofilm formation abilities of Y-TZP and PEEK were lower than that of Ti-6Al-4V; therefore, the sensitivities to PDT and AMS were higher, and the biofilm removal abilities were also conspicuous (Figure 6). The results of the PEEK in the present study also indicated that *S. mutans* was more effective than *A. actinomycetemcomitan*. Among all the samples, Y-TZP exhibited the smoothest surface. However, the attachment of the *A. actinomycetemcomitan* dental plaque needs to rely on other strains. Herein, *A. actinomycetemcomitan* was difficult to directly attach on the Y-TZP surface. Thus, the biofilm removal ability of *A. actinomycetemcomitan* through either PDT or AMS was better than that of *S. mutans* in the Y-TZP group.

The merits of PEEK, such as hydrophobicity and chemical inertness, may prove as disadvantages for the application of PEEK to dental implantology. These points would negatively impact cell adhesive effects, bone attachment, and osseointegration. The human osteoblast-like (MG-63) cells are commonly used osteoblastic models that are used to study bone cell viability, adhesion, and proliferation on the surfaces of load-bearing biomaterials [6]. Ti-6Al-4V and its compounds are gold standard materials for implants or abutments owing to their excellent mechanical strength, chemical stability, biocompatibility, and high osseointegration ability [6,12]. Martins et al. found that the bone response to Y-TZP implants is comparable with that observed around Ti-6Al-4V implants [12]. The present study did not conduct in vivo experimentation, but the analysis of the cell viability (MTT test) of the PEEK and MG-63 cells in this study (Figure 7) suggested that PEEK yields equivalent cell viability responses to those of Ti-6Al-4V or Y-TZP (*p* > 0.05). These findings indicate that PEEK also possesses good biocompatibility for MG-63 cells. With the combination of the verification results from this study and the data reported in the literature [42,43], it can be summarized that PEEK has anti-adhesive bacterial properties. With comparable MG-63 cell viability to Ti-6Al-4V and Y-TZP, PEEK is a suitable material for dental implants or abutment from a microbiological aspect.

The PAEK family has been increasingly employed as biomaterials for orthopedic, trauma, and spinal implants. In addition to PEEK, other PAEK family members, such as poly-ether-ketones or poly-ether-ketone-ketone (PEKK), are considered for dental implant or abutment materials [34]. Our previous study had investigated the bonding properties of two PAEKs materials (i.e., PEEK and PEKK) [44]. Within the experiment, the surface roughness, morphology, and hydrophilicity of the two PAEK materials were measurement and compared [44]. The results indicated that no significant difference could be found between the surface roughness and hydrophilicity of the two PAEK materials after process grinding surface pre-treatment. Yuan et al. [45] compared the osteointegration property between PEKK and PEEK; the report indicated that the other ketone group in PEKK increases the ability of surface chemical modification. This might affect the cell behavior and osteointegration of PEKK. Additionally, the PEEK material used in this work can be processed via various digital workflow like computer-aided design and computer-aided manufacture or additive manufacture system; nonetheless, commercial PEKK materials can only process via the former. However, in future dental practice, dental implants or abutments are urgently needed to be manufactured through customization; therefore, only PEEK materials were discussed in this work.

The present study showed that the cell viability of MG-63 on PEEK is similar to those of other materials (Ti-6Al-4V and Y-TZP). As mentioned previously, the biofilm formation ability was related to hydrophilicity, surface roughness, and surface free energy. Although the present in vitro study did not consider PEKK, according to the previous report [42,43] it can be predicted that PEKK will have similar biological characteristics as PEEK. However, whether PEEK affects osteoblasts and their differentiation needs additional investigation. In the future, it is important to increase the bacterial photosensitizer absorption rates and penetrating abilities and to develop better light sources for PDT to reduce exposure time and to make it more convenient for use in treatments in the dental clinic.

## 5. Conclusions

Poly-ether-ether-ketone (PEEK) was significantly less conducive to biofilm formation compared with titanium alloys (Ti-6Al-4V) and had comparable MG-63 osteoblast cell viability to the Ti-6Al-4V and zirconia-based ceramic materials (Y-TZP). Photodynamic therapy (PDT) had similar antimicrobial efficacy and exerted biofilm removal effects to antibiotics. Therefore, it is inferred to be an effective and substitute therapeutic approach for peri-implant mucositis. A comprehensive consideration of the results obtained from this in vitro study indicates that PEEK is a possible alternative material for dental implants or abutments. When peri-implant mucositis occurs, PDT may be a suitable therapeutic approach, especially for patients with allergic reactions to antibiotics.

## Figures and Tables

**Table 1:** Dental materials used in this study.

Materials	Abbr.	Main Composition ^1^	Manufacturer
Alloy material			
ASTM F136	Ti-6Al-4V	Ti, Al, V, others	Green Dentech Co. Ltd., Tainan, Taiwan
Ceramic material			
90X10-HT	Y-TZP ^2^	ZrO_2_, Y_2_O_3_, others	Aidite Technology Co., Ltd., Qin Huang Dao, Mainland China
Polymer material			
VESTAKEEP	PEEK ^3^	Poly-ether-ether-ketone	Evonik Japan Co., Tokyo, Japan

^1^ According to the information provided by the manufacturer. ^2^ Y-TZP, yttria-stabilized tetragonal zirconia polycrystal. ^3^ PEEK, poly-ether-ether-ketone.

## Data Availability

The data presented in this study are available on request from the corresponding author.
